# Religious‐Tailoring of Mental Health Services in Ontario: A Qualitative Study Exploring Service User and Provider Recommendations

**DOI:** 10.1002/jcop.70036

**Published:** 2025-09-04

**Authors:** Syed Faruk, Muhammad Asadullah, A. Ka Tat Tsang

**Affiliations:** ^1^ Faculty of Social Work University of Toronto Toronto Ontario Canada; ^2^ Department of Justice Studies University of Regina Regina Saskatchewan Canada

**Keywords:** faith‐based, mental health service, muslim community psychology, psychospiritual, psychotherapy, religious tailoring

## Abstract

Aims

This study aimed to explore the experiences of Muslim mental health service users and providers in Ontario, Canada. With a focus on understanding mental health service barriers and facilitators, the research also sought to identify service user and provider recommendations and highlight the capacity for addressing gaps within service utilization and delivery by adopting religious tailoring with the Muslim community. This was carried out through a CBPAR approach to conduct diverse focus groups with Muslim service users and service providers across Ontario, Canada. The results indicate mistrust towards Eurocentric models, cultural stigma, and insufficient provision of religiously and culturally congruent care significantly hinder engagement. Nevertheless, religious tailoring—incorporating elements such as Imams, faith‐based counseling, and community‐based interventions—emerges as an effective strategy to overcome these challenges. The study powerfully emphasizes the success of integrating mental health services within mosques and Muslim‐led organizations, equipping Imams with skills in psychospiritual counseling, and weaving Islamic coping mechanisms into therapeutic practices. The study concludes with 11 practical recommendations based upon service user and provider suggestions that can empower policymakers, practitioners, and Muslim communities to enhance mental health accessibility and encourage meaningful engagement.

## Summary

- Community‐based and psychology‐related:
  ◦ This study is the largest qualitative study utilizing community based participatory action research methods with the Muslim community.
  ◦ It adds to the literature, religious tailoring recommendations made by service users for psychological and mental health programming and providers.
- Approach proves advantageous; will be contingent upon the individual service user's lived experiences.
- Represents the most extensive qualitative analysis of mental health within the Muslim community.
- Practicable to implement across a multitude of mental health service and provider relationships.

## 1 Introduction

Muslim community members consistently identify the accessibility of mental health services as well as heightened psychological distress encompassing anxiety, depression, and posttraumatic stress disorder (PTSD), as critical concerns (Chaudhary et al. 2019; Amri and Bemak 2013). Nevertheless, empirical research indicates a notable underutilization of these services or a less favorable disposition toward mental health care (Aloud and Rathur 2009; Ali et al. 2022; Ahmed and Mao 2024). This phenomenon constitutes a social issue that necessitates accountability. In the United States, researchers utilized a nationally representative sample and discovered that, although the prevalence of psychiatric disorders among Muslims was similar to that of non‐Muslims, Muslims were significantly less likely to seek help and faced greater challenges in social and emotional functioning. Similar inhibitions have been documented in Canada (Reich et al. 2024) and the United Kingdom (Tannerah et al. 2024). Consequently, there exists a disjunction between Muslim community members suffering from mental health disorders and the services that have been established to address their needs, which warrants further investigation. This study employs a triangulation approach to review findings from Canada, the United Kingdom, and the United States to synthesize literature on the barriers and facilitators of mental health services within the Muslim community. The selection of these three nations was deliberate to achieve the maximal depth possible, as they have collectively produced the most number of English studies pertaining to this subject among countries with non‐Muslim majority populations. Although beyond the scope of our literature review, many of the findings have also been observed in other Western or non‐Muslim majority countries (refer to McLaren et al. 2023 or Omar et al. 2017 for examples from Australia and Pooremamali et al. 2012 for a study conducted in Sweden). It ultimately serves as a basis for presenting Ontario as a case study for effective mental health service facilitation, as advocated by voices from the Muslim community.

## 2 Literature Review

Religious concerns play a critical role, although there are often gray areas where cultural and religious beliefs overlap (Ahmed and Mao 2024). In the United States, stronger adherence to cultural and traditional explanations of mental health problems correlated with less favorable attitudes toward formal mental health services (Khan et al. 2019). Studies from Canada (Ahmed and Mao 2024; Al‐Janaideh et al. 2023; Reich et al. 2024; Elkassem and Csiernik 2020; Ibrahim and Mojab 2023; Zia and Mackenzie 2024), the United Kingdom (Mahmud 2024; Tannerah et al. 2024; Siebert and Souto‐Galvan 2024; Pilkington et al. 2012; Weatherhead and Daiches 2010) and the United States (Aloud and Rathur 2009; Ali et al. 2022; Amri and Bemak 2013) indicate that Muslim attitudes towards mental health services are influenced by religious values, past experiences—including instances of human rights violations—the inability to form therapeutic relationships with incompetent practitioners, and mistrust of healthcare providers aligned with Eurocentric models that marginalize their identities. These factors collectively discourage engagement. Further, while existing studies have found that therapists must acknowledge clients' religious frameworks to build trust (Reich et al. 2024 and Tannerah et al. 2024), the results of our study along with the subsequent discussion will strengthen the existing body of literature by addressing the nuances of service provider development needs and the integration of religious values as potential strategies to mitigate these barriers.

### 2.1 Solutions

The role of faith leaders such as Imams, Shaikhs and community leaders is a common theme. Studies in Canada (Ahmed and Mao 2024; Elkassem and Csiernik 2020; Ibrahim and Mojab 2023; Albatnuni and Koszycki 2020; Gokani et al. 2023), the United Kingdom (Khalifa et al. 2011; Tannerah et al. 2024; Siebert and Souto‐Galvan 2024; Ozeto and Allan 2021; Meran and Mason 2019; Weatherhead and Daiches 2010) and the United States (Aloud and Rathur 2009; Amri and Bemak 2013; Khan et al. 2019; Ali et al. 2022; Suleiman et al. 2023; Humam et al. 2023) have found that Imams in mosques frequently served as informal counselors and crisis responders. They also note a strong preference among Muslims for religiously aligned mental health support through spiritual healers as well as a preference for informal methods of healing, such as family support, community support or religious intervention over formal mental health services. Ibrahim and Mojab (2023) found that this was especially true in cases involving domestic violence, youth delinquency, and intergenerational conflicts among Muslims in Vancouver, British Columbia, Canada.

Furthermore, Muslim women emphasized the importance of gender‐sensitive approaches. For example, Muslim women in the United States (Humam et al. 2023) have advocated for the inclusion of female religious leaders to address issues specific to their community. Conclusively, literature often stresses the role of Imams as trusted figures in bridging the gap between communities and mental health services.

There is a trend in the United States (Suleiman et al. 2023; Awaad, Midani et al. 2024; Humam et al. 2023; Syed et al. 2020) for training local Imams and community leaders with the psychospiritual tools that they need for responding to mental health issues on the frontline. Collaborations among mental health service providers, Imams and Muslim community organizations have co‐developed and implemented crisis response manuals and training for Islamic clergy. Implementation is also present in Canada to a significantly lesser extent (Syed et al. 2020), although it has been called for in multiple studies (Reich et al. 2024; Elkassem and Csiernik 2020; Ibrahim and Mojab 2023; Hunt et al. 2020). Similar recommendations have been advocated for by researchers in the United Kingdom (Tannerah et al. 2024; Meran and Mason 2019). Future endeavors are soon to demonstrate controlled experimental testing of such interventions in this promising mental health service innovation.

While Western literature increasingly highlights the value of training Imams in mental health response, it seldom centres Imams as primary informants or examines the moral, legal, and structural tensions shaping their roles. Most studies utilizing the perspectives of Imams rely on small samples (Ibrahim and Mojab 2023), a limited number of Imam participants (Humam et al. 2023) or post‐training surveys (Syed et al. 2020). In contrast, research from majority Muslim countries offer a more policy‐attuned and theological lens. Islam et al. (2024) surveyed 162 Imams in Bangladesh and found only 2.47% correctly rejected the myth that most people who die by suicide are psychotic, thus highlighting critical literacy gaps. Arafat et al. (2024) reported that while 62% of Imams in their study believed that suicide attempt survivors need help and supported hospital referral, half of the participants' perceptions still opposed decriminalization of suicide, perhaps revealing ideological ambivalence. These studies underscore that Imams are not just training recipients but complex actors shaped by religious and legal frameworks as well as moral reasoning. To be effective, Western training models must move beyond discussions surrounding literacy and engage more deeply with the policy and theological discourses that inform Imam behavior—particularly on stigmatized issues like suicide.

Research conducted in Canada (Kennedy‐Turner et al. 2023; Al‐Janaideh et al. 2023; Moscovitz et al. 2023; Reich et al. 2024; Alghamdi et al. 2022; Ashbourne et al. 2021; Gokani et al. 2023), the United States (Awaad, Midani et al. 2024; Amri and Bemak 2013; Aloud and Rathur 2009), and the United Kingdom (Mahmud 2024; Jabeen and Snowden 2022; Tannerah et al. 2024; Siebert and Souto‐Galvan 2024; Weatherhead and Daiches 2010) have collectively advocated for the implementation of cultural sensitivity training for healthcare personnel; the incorporation of Islamic practices into mental health services and interventions; and/or the diversification of mental health services through the recruitment of Muslim mental health practitioners. It is imperative that providers exhibit transparency regarding their limitations, whilst simultaneously circumventing unconscious biases that may be erroneously construed as professional deficiencies (Moscovitz et al. 2023). Muslim individuals residing in Quebec, Canada, have expressed that service providers often lack the requisite skills to address the specific needs of Muslim women effectively. As a result, these women are frequently compelled to educate their therapists about their cultural and religious backgrounds. This circumstance contributes to an emotional strain to the therapeutic process and serves to deter ongoing engagement with services (Reich et al. 2024).

Incorporating Islamic practices such as ruqya (healing intervention), prayer, reflection on the Quran and Hadith, meditation on the 99 names of God, and/or du'a (supplication) into mental health services or therapy sessions may enhance engagement and satisfaction among participants. This is particularly relevant as Muslims in Canada (Reich et al. 2024; Elkassem and Csiernik 2020; Ibrahim and Mojab 2023; Hassan et al. 2021; Albatnuni and Koszycki 2020; Jozaghi et al. 2016; Gokani et al. 2023), the United Kingdom (Mahmud 2024; Jabeen and Snowden 2022; Ozeto and Allan 2021; Weatherhead and Daiches 2010; Ally and Brennan 2015), and the United States (Ali et al. 2022; Humam et al. 2023) have identified religious faith as a significant source of support, resilience and coping. Interestingly, Wu and Schimmele (2021) used data from the nationally representative 2014 General Social Survey (GSS) in Canada and found that frequent participation in religious services and the importance of spirituality buffered the negative effects of religious discrimination on mental health. Similarly, Ottawa‐based researchers Albatnuni and Koszycki (2020) found that salah (obligatory prayer) was positively associated with subjective well‐being. In addition to religious coping, Muslims in Canada discussed self‐management strategies such as engaging in physical activities and attending educational classes (Ahmed and Mao 2024). This highlights the need identified in studies for the development of self‐help resources that incorporate Islamic teachings to encourage self‐care and mental health management (Reich et al. 2024). However, as noted by Ozeto and Allan (2021), religious coping mechanisms may be significantly helpful only for individuals with higher levels of religiosity.

Embedding mental health services within culturally specific facilities and outreach programs to reduce barriers, increase utilization, and improve mental health outcomes for Muslims has been widely recommended, including in Canada (Elkassem and Csiernik 2020; Ibrahim and Mojab 2023; Hassan et al. 2021; Hunt et al. 2020; Ashbourne et al. 2021; Gokani et al. 2023), the United Kingdom (Tannerah et al. 2024; Siebert and Souto‐Galvan 2024; Weatherhead and Daiches 2010) and the United States (Awaad, Hussein et al. 2024; Khan et al. 2019; Chaudhary et al. 2019; Amri and Bemak 2013). It is important to note that the Hunt et al. (2020) study, which utilized semi‐structured interviews with Muslim women in Waterloo, Ontario, Canada, found that some participants highlighted intra‐community discrimination and exclusionary practices within community spaces (e.g., favoritism or ethnic divides) as factors limiting accessibility and usefulness. Black Muslim women, in particular, reported feeling marginalized even within Muslim community spaces, where they faced anti‐Black racism that further compounded their sense of isolation. Thus, it is incumbent upon Muslim community leaders and members to champion psychological advancement and social inclusion, aligning these efforts with the rich legacy of the community.

## 3 Methods

This study adopted a Community‐Based Participatory Action Research (CBPAR) framework to prioritize community involvement and empowerment. CBPAR fosters reciprocity between researchers and community stakeholders, aligning the research with community priorities (Maiter et al. 2008). The implementation of CBPAR involved:

1. Steering committee: Composed of scholars and representatives from Muslim‐led organizations, the committee guided the research, critiqued materials, and contributed to data analysis. This approach emphasized shared ownership and co‐creation of knowledge.
2. Peer researchers: Four Muslim community members with lived experiences of accessing social services were recruited as paid peer researchers. They contributed to data collection and analysis, enriching the study's cultural relevance and community engagement.
3. Collaborative research sessions: Four sessions were conducted to involve stakeholders in developing research questions and analyzing data, ensuring that findings were grounded in community experiences.
4. Capacity development: Community stakeholders participated in research training seminars to enhance their skills and promote sustainable research capacity within the community.

As members of the Muslim community and Muslim scholars, our methodology profoundly aimed to illuminate the understanding of mental health services within the Muslim population by investigating whether participants' responses resonate with Islamic principles, such as advocating for the healing of mental illness or providing support to those in need, as socially instructed by the Prophet, peace be upon him. Furthermore, we delved into the meanings participants attribute to mental health services.

The recruitment strategy aimed to reflect the diversity of Ontario's Muslim population. Service users were recruited using a combination of digital advertisements, in‐person efforts, and community collaborations. Materials were translated into Arabic and Dari and disseminated through partner organizations. Eligibility criteria for service users included: (1) being over 16 years of age, (2) self‐identifying as Muslim, (3) residing in Ontario, and (4) having accessed social services within the past 2 years. Service provider recruitment relied on community partner networks. Eligibility criteria for service providers included: (1) being over 18 years of age, (2) self‐identifying as Muslim, (3) residing in Ontario, and (4) having delivered social services to Muslim clients within the past 2 years. Targeted recruitment for diversity was enhanced by partnerships with organizations such as the Rohingya Centre of Canada and Naseeha Mental Health, which ensured representation across racial, linguistic, and geographic backgrounds.

### 3.1 Data Collection

Data were collected through focus groups with service users and providers, exploring their experiences with social services. Protocols, collaboratively developed and approved by the Steering Committee, included semi‐structured interviews that allowed for open‐ended discussion and targeted inquiry. Materials were translated into Arabic and Dari to accommodate linguistic diversity. A total of 12 focus groups were conducted with service users, and three with service providers. Nine focus groups were conducted in English, two in Dari, and one in Arabic. Service user focus groups were stratified by gender and life stage, reflecting the study's intersectional focus. After the focus group, participants completed an online demographics questionnaire using Qualtrics, a secure survey platform, to provide contextual data for analysis.

### 3.2 Data Analysis

Audio recordings were transcribed and, where applicable, translated into English by peer researchers. Thematic analysis was used to identify patterns and themes within the data, following Braun and Clarke's (2006) framework:

1. Understanding: Researchers immersed themselves in the data to grasp participant experiences.
2. Synthesizing: Team discussions identified recurring ideas and patterns.
3. Theorizing: Themes and coding frameworks were collaboratively developed with the Steering Committee across two collaborative research sessions.
4. Re‐contextualizing: Findings were contextualized within existing literature and theoretical frameworks.

Research assistants and peer researchers were trained in NVivo 12 software and thematic analysis, ensuring consistent and reliable coding. Simulated exercises refined coding frameworks and enhanced inter‐coder reliability. Regular team meetings and feedback from the Steering Committee guided ongoing analysis and the planning of subsequent interviews. Research investigators and assistants identified 11 practical steps recommended by focus group participants that align with the themes in our results. The Steering Committee guided the extraction process, involving the input of Imams and Scholars to enhance the action steps proposed by the focus groups by providing specific practical examples of Islamic teachings for service providers and agencies to follow. This study constitutes a component of a broader initiative exploring social services within the Muslim community; nonetheless, this manuscript exclusively concentrates on themes pertinent to mental health services.

### 3.3 Position Statement

The findings from this study project were part of a larger study funded by the Social Sciences and Humanities Research Council (SSHRC), aimed at understanding the social service needs of Muslim communities in Ontario, Canada. This study boldly expands the prevalent Eurocentric perspectives that have historically dominated mental health studies, aligning with de Sousa Santos (2012) epistemology of the south, while also emphasizing the urgent need for a rich tapestry of diverse experiences and intercultural dialog. In light of this, our research is profoundly anchored in the hadith of Al‐Bara' bin' Azib (May Allah be pleased with them), who conveyed: The Messenger of Allah, peace be upon him instructed us to engage in seven things: visiting the sick, attending funerals, invoking Allah's Mercy upon those who sneeze (i.e., by saying: Yarhamuk‐Allah), supporting the vulnerable, aiding the oppressed, promoting the greeting of ‘As‐Salamu ‘Alaikum’, and to help those who swear to do something to keep their oaths (Al‐Bukhari and Muslim). This was meticulously recorded by Imam Al‐Nawawi in Hadith #846 of Riyadh as‐Salihin (1277). This study also firmly grounds itself in intersectionality theory, acknowledging how interconnected social categories—such as race, class and gender, ability, citizenship, sexual orientation, and religion—profoundly shape both access to and experiences with social services (Crenshaw 1989). It powerfully illustrates how the interplay of multiple marginalized identities can significantly influence interactions with health services (Bauer 2014). Furthermore, the study incorporates the intersectional invisibility hypothesis, which compellingly posits that individuals possessing multiple marginalized identities encounter distinct challenges, including a lack of recognition and representation (Purdie‐Vaughns and Eibach 2008). Our unique framework was implemented through a collaborative approach that embraced the diverse perspectives of the Muslim community during the entire research process, firmly rooted in our collective prophetic guidance to support the vulnerable. In our study, we aimed to empower the underrepresented voices of Muslims within mental health services and intentionally engaged a wide range of marginalized Muslim identities. Moreover, we harnessed these vital voices to shape our research questions, foster community engagement, and conduct data collection and analysis, all of which were facilitated by our carefully chosen study methodology.

### 3.4 Ethical Considerations

The University of Toronto's Research and Ethics Board approved the study, which prioritized participant confidentiality and informed consent. Safeguards included random numerical identifiers, exclusive use of audio recordings, and guidelines to avoid personal identifiers during focus groups. All authors have no conflicts of interest to report.

## 4 Results

The findings begin with participants' (service users and providers) reports of a lack of culturally and/or religiously sensitive mental health services, followed by service user‐proposed solutions/recommendations and, finally, service provider solutions/recommendations for tailoring mental health services to the needs of the Muslim community. All quotes are verbatim, except for minor changes to demographic information to protect participant anonymity.

### 4.1 Lack of Culturally and/or Religiously Sensitive Services

The predominant concern articulated by both service recipients and providers was a “lack of culturally and/or religiously sensitive services.” Participants emphasized the lack of mental health provisions specifically designed for the Muslim population.

*“So, I know that we've talked about mental health a lot, so that's one big thing that probably needs to be like more hyper‐focused towards Muslims.”* (Male Youth Focus Group 1, Pt #458)

*“Something else that came to mind was counseling services, especially affordable ones. The lack of…culturally and religiously competent training…There might be accessible counseling available for people, but it may not be culturally and religiously sensitive.”* (Female Youth Focus Group 1, Pt #429)

Service providers have additionally underscored this impediment:

*“They don't wanna go through explaining to their therapist, or whatever about the cultural identity or the spiritual identity…they may be like, oh, I may get made fun of, or that they may not understand.”* (Service Providers Focus Group 2, Pt #422)

This theme emerged in 45 instances and aligns with conclusions drawn in existing scholarly literature. The following section outlines suggestions for addressing these obstacles.

### 4.2 Recommendations for Religious Tailoring: Service Users

Service users articulated three principal methodologies for religiously tailoring mental health services: (1) understanding cultural practices, (2) integrating faith‐based counseling, and (3) employing community‐centric approaches.

### 4.3 Theme 1: Understanding Cultural Practices

Participants advocated for services to incorporate Islamic values, traditional practices, and cultural subtleties. Practitioners ought to possess an understanding of cultural conventions pertaining to modesty, family roles, and gender interactions.

*“When it comes to mental health, I feel like there's a lot of layers…if there's like an understanding…maybe spirituality, it could help that person more…maybe a social worker who's also from the same background as you or same religion, or like, has the same viewpoints, or understanding as you.”* (Female Youth Focus Group 2, Pt #461)

*“…and find people who can counsel. If I am sharing my experiences…I don't want your sympathy, I want you to show me empathy…someone who has already been through what I'm going through. That's how you can fix me.”* (Male Adult Focus Group 3, Pt #701)

*“…counselor and he's a male…that's just kinda awkward…that gives me hesitation like, ‘Okay never mind I don't want to go talk to them anymore.’”* (Female Youth Focus Group 3, Pt #508)

This theme, referenced on 14 occasions, encompasses the imperative of eschewing cultural stereotypes and engaging service users as distinct entities situated within their respective cultural frameworks.

### 4.4 Theme 2: Integration of Faith‐Based Counseling

Participants frequently endorsed the necessity of faith‐based counselors trained in both Islamic theology and modern psychotherapies.

*“Let's say prayer or whatnot…incorporating that in their advice…mental health services…maybe you could incorporate your spiritual beliefs into…healing…so that there's like more of an impact…like it's more personal.”* (Female Youth Focus Group 2, Pt #461)

*“When it comes to these guys (providers), they don't have knowledge. We need someone with knowledge of the deen, number one, a Muslim, who is a practicing Muslim that has a grounded knowledge of Islam and not a secular knowledgeable about Islam.”* (Male Youth Focus Group 4, Pt #578)

*“…people who have knowledge of the world, who are well educated in Din as well as in Duniya…should come forward…”* (Male Seniors Focus Group 1, Pt #569)

This theme appeared 15 times, suggesting a strong consensus among participants about the need for culturally conducive providers who can bridge both religious and worldly knowledge.

### 4.5 Theme 3: Community‐Centric Approaches

Participants underscored the significance of mosques, community centres, and religious associations in fostering partnerships with mental health services and support systems.

*“It has to be not a one‐man show. It should be the community program…You tell your problem, and you listen. You lend the compassionate ear to other peoples' problems. It helps them. It maybe can also help on a one‐on‐one basis.”* (Female Seniors Focus Group 1, Pt #515)

*“You know our Imams and Muslim scholars…if we can involve those people…it will be very much helpful.”* (Male Seniors Focus Group 1, Pt #529)

*“…like in the masjid…there could be a support there.”* (Female Youth Focus Group 3, Pt #504)

This theme appeared 12 times, highlighting the importance of community involvement and the potential for faith‐based initiatives to create a supportive environment for individuals facing challenges.

### 4.6 Recommendations for Religious Tailoring: Service Providers

Service providers proposed three primary methodologies: (1) access to culturally specific services, (2) holistic services for addictions, and (3) leveraging the role of community and mosques.

### 4.7 Theme 1: Access to Culturally Specific Services

Similar to the perspectives of service users, service providers also emphasized the need for services that address the distinct requirements of the Muslim population, including the involvement of providers who understand Islamic values and cultural contexts.

*“…a lot of people I work with are families…immigrants and newcomers to Canada…looking for services that are Muslim and…culturally appropriate for them…but…they don't really exist.”* (Service Provider Focus Group 1, Pt #421)

“*Speaking as a mental health counselor at Naseeha lines…there's this added layer of cultural and spiritual trauma that I'm noticing is the bigger difference in Muslim youth and families where it's like you know what does culture say about certain things…then there's that bigger piece of how do I fit in my spirituality into all of this…Especially in the Western world where you're constantly it seems balancing two identities.”* (Service Provider Focus Group 2, Pt #441)

*“…lack from the mental health space…one of the biggest barriers is a lack of psychological safety…You know they may have already felt pushed away by many parts of the community, and that's why they may be seeking culturally‐sensitive care…if that is not integrated when their expectation is that it is, I think that puts a lot of people off.”* (Service Provider Focus Group 4, Pt #546)

This theme was mentioned 21 times, highlighting the critical need for mental health services that prioritize inclusivity and understand diverse cultural backgrounds.

### 4.8 Theme 2: Holistic Services for Addictions

Participants underscored the importance of addressing addiction issues in conjunction with other needs such as family support and community integration.

*“I'm noticing a big kind of need for addiction services recently…A lot of callers are calling in with needs that cater to those things.”* (Service Provider Focus Group 2, Pt #441)

*“…we provide addictions care…But 95% of the help…is interfacing with social work…helping them feel supported…maybe a conversation about depression…”* (Service Provider Focus Group 4, Pt #545)

Nonetheless, the process of community integration may be hindered by elements of social acceptability.

*“Muslims tend to stay away because our…we're a bit biased with that…So I know there are a few places which have supervised injection sites…but I know Muslims would try to avoid that particular agency.”* (Service Provider Focus Group 1, Pt #436)

Moreover, there exists a notable deficiency in the development of culturally attuned and holistic services or networks within the Muslim community.

*“Sometimes we don't have those structures in place or enforced for us to actually be able to get to that part.”* (Service Provider Focus Group 2, Pt #422)

This theme was mentioned eight times, highlighting the urgent need for tailored outreach programs that foster recovery while strengthening individuals' connections to their community, thereby creating a supportive environment that promotes lasting change.

### 4.9 Theme 3: Role of Community and Mosques

Mosques were identified as potential hubs for mental health services, enhancing both counseling and education in a trusted setting.

*“…professionals who are well‐versed…collaborate with…Imans…therapists or like psychologists and figure out and understand…have a mutual understanding.”* (Service Provider Focus Group 2, Pt #422)

*“…mental health context into some prophetic stories, such as even the Prophet (sallallahu alaihi wasallam) lost someone or even Prophet Isa (alayhis salam) was raised by a single parent…we had this really beautiful training session once where an Imam came and recontextualized Surah Al‐Duha as a framework of how to help someone during intense grief…really beautiful recontextualization.”* (Service Provider Focus Group 4, Pt #545)

Service providers additionally articulated the constraints associated with the collaboration of non‐Muslim organizations with mosques.

*“…it's difficult to get them to trust us as well, and to refer…we're not specifically tailored to Muslims, maybe that's the problem.”* (Service Provider Focus Group 1, Pt #439)

This theme was mentioned 14 times, highlighting solutions with ongoing challenges faced in bridging the gap between faith‐based services and broader community support systems.

## 5 Discussion

The results of the present study emphasize the urgent need for mental health services that are culturally and religiously tailored to the specific needs of the Muslim population. Both service recipients and providers expressed significant concerns about the lack of such services, aligning with existing literature on the barriers that Muslims encounter in accessing appropriate mental health care (Reich et al. 2024; Mahmud 2024; Humam et al. 2023). Although emerging research sheds light on methods for religious customization—such as Awaad, Durrani et al. (2024) and Awaad, Hussein and colleagues' (2024) model for suicide response and community healing, along with a corresponding training manual (Awaad, Midani et al. 2024), and a continuum of approaches with traditional Islamically integrated therapies (Khan et al. 2025) on one end and Islamically modified Western therapeutic approaches (Qasqas 2024) on another,—there remains a lack of literature delineating specific, actionable strategies for tailoring services beyond suicide intervention or psychotherapeutic frameworks. This deficiency is of paramount importance, given that most mental health services do not exclusively concentrate on suicide prevention or lack the capacity to implement specialized therapeutic modalities. The findings of the present study help address this gap by outlining essential service recommendations: understanding cultural practices, integrating faith‐based counseling, adopting community‐centric approaches, and providing holistic addiction services.

Service users emphasized the importance of understanding cultural practices, while service providers focused on promoting access to culturally specific services. Despite both themes acknowledging the necessity for cultural sensitivity, there are noticeable differences in how they are presented. For instance, service users expressed their feelings of alienation to support this need, whereas service providers seemed to accept this need without further exploration, moving on to the challenges that arise, including the limited availability of services capable of meeting this need or their own limitations as Muslim mental health service providers. Service users also suggested integrating faith‐based counseling, but while service providers often acknowledged the significance of Islamic knowledge in their services, it did not emerge as one of their top three priorities. This might have been missed by Muslim service providers who work within Muslim mental health service settings, considering that they may themselves partially fulfill the service gap emphasized by service users. However, service providers did recommend holistic services for addiction, which service users did not mention. Although this discrepancy is not entirely clear, it might be explained by service providers' observations of stigma and shame related to addiction and recovery, suggesting that service users could have been hesitant to share their substance use issues in group discussions with peers. Service providers also noted that structural barriers marginalizing Muslim mental health service users might hinder their ability to “get to that part” of their journey of recovery. Interestingly, this could complicate some clinical presentations, such as cases where sometimes symptoms of psychiatric disorders like PTSD are masked by substance use (Petrakis et al. 2002) or situations where chronic substance use leads to cortical brain changes contributing to cognitive dysregulation during abstinence (Bernheim et al. 2016). This underscores the need for integrative or holistic methods. Finally, service users advocated for community‐centric approaches, while service providers suggested strengthening the role of the community and mosques. These themes largely convey similar ideas, highlighting essential roles. Based on their experiences, service providers shared additional insights, including personal stories of how their service delivery has benefited from such underused collaborations in the past, as well as the collaboration challenges faced by even Muslim service providers working in service settings that are not tailored specifically for Muslims, which can result in a lack of cultural safety.

Practical steps for the attainment of these solutions were derived from focus groups and are further substantiated by literature:

1. Using prophetic stories: The lives of Prophets and companions serve as powerful parables for coping with adversity. The Prophet Muhammad (peace be upon him) himself used such stories for consolement. For instance, when the Prophet (peace be upon him) was falsely accused, Ibn Mas'ud (may Allah be pleased with him) reported that the Prophet's face became red with emotion, and he said, “Who will do justice if Allah and His Messenger do not?” He then said, “May Allah have mercy on Musa; he was caused more distress than this but remained patient” (Al‐Bukhari and Muslim). As documented by Imam Al‐Nawawi in *Hadith #42* of *Riyadh as‐Salihin* (1277). Practitioners can integrate such stories into therapy to help Muslim clients navigate distress in a culturally and religiously relevant way.
2. Integrating Quran and Hadith teachings: The Quran and Hadith offer valuable lessons. Owens et al. (2023) scoping review highlights mental health interventions incorporating Quranic guidance, providing a basis for additional inquiry.
3. Incorporating supplications (Dua): Supplications (duas) play a pivotal role in fostering resilience, promoting well‐being, and transforming maladaptive cognitive patterns. For example, the Prophet (peace be upon him) said “Let not one of you wish for death because of a misfortune which befalls him. If he cannot help doing so, he should say: ‘O Allah, keep me alive as long as You know that life is better for me, and make me die when death is better for me” (Al‐Bukhari and Muslim). This powerful supplication, documented by Imam Al‐Nawawi in *Hadith #40* of *Riyadh as‐Salihin* (Al‐Nawawi 1277), serves as a practical tool for managing suicidal thoughts and fostering spiritual resilience. Incorporating daily and situational supplications, as emphasized by Latif et al. (2024, 75–80), can help individuals combat distress and maintain emotional equilibrium.
4. Encouraging Salah (five daily prayers): In addition to its spiritual advantages, Salah may enhance social connections and emotional support when accompanying mosque attendance. However, practitioners should consider religiosity and life stages (Nguyen et al. 2013). Nonpracticing Muslim therapists should also be mindful of potential guilt or transference issues, particularly in light of Quranic verses cautioning against hypocrisy (Surah As‐Saf 61:2–3). “O you who believe! Why do you say that which you do not do? Most hateful it is with Allah that you say that which you do not do” (compiled by Imam Al‐Nawawi in the beginning of chapter 24 of *Riyadh as‐Salihin*, 1277).
5. Understanding cultural nuances: The diversification of the workforce has the potential to effectively address cultural nuances. It is recommended that non‐Muslim practitioners recognize and respect Muslim cultural frameworks (Aloud and Rathur 2009), refrain from perpetuating stereotypes, and maintain an awareness of their own unconscious biases (Moscovitz et al. 2023). It is plausible that Muslim practitioners may be afforded greater leniency by service users regarding theological discrepancies compared to their non‐Muslim counterparts, particularly considering the pervasive mistrust of the mental health service system that has been reported by participants and corroborated by prior research (Ibrahim and Mojab 2023).
6. Gender sensitivity: Approaches that are specific to gender demonstrate efficacy in culturally customized mental health interventions. For instance, the trauma intervention conducted by Zoellner et al. (2018) employed facilitators of the same gender and incorporated Islamic teachings, leading to a significant diminishment in both the severity and symptoms of PTSD.
7. Theological and psychological training: Professionals may not possess the requisite skills to thoroughly engage with Islamic theological discourse. The formulation of succinct, disorder‐focused educational modules via Islamic institutions has the potential to rectify this deficiency, analogous to the approaches observed in Western programs (Tse 2002).
8. Community and mosque collaboration: Collaborations such as those established between the Khalil Centre and our community partner, the Scarborough Muslim Association, which provided psychotherapist‐facilitated support groups for individuals who have experienced divorce, or the mosque‐based community education initiative developed by Chaudhary et al. (2019) that employed cultural framing to address manifestations of PTSD, exemplify the potential inherent in community‐centred methodologies. Nonetheless, these initiatives frequently encounter obstacles related to sustainability, primarily due to constraints in funding. It is imperative that policymakers and funding agencies extend their support to these collaborative efforts, while mosques should take the initiative to actively pursue partnerships.
9. Mosque‐approved services: Mosques should maintain lists of approved therapists and services, especially in metropolitan areas. Rural regions, however, may face significant limitations due to fewer Muslim‐specific resources.
10. Community‐based psychoeducation: The effectiveness of religiously tailored initiatives is contingent upon the mitigation of stigma and the cultivation of supportive environments conducive to the flourishing of services (Hunt et al. 2020).
11. Holistic addiction services: Effective addiction interventions, such as the mosque‐based psychoeducation program implemented by Hassan et al. (2021), amalgamated Islamic precepts with empirical evidence, resulting in heightened awareness and substantial user satisfaction. Similarly, the smoking cessation initiative conducted by Pratt et al. (2021) strategically utilized the month of Ramadhan and incorporated religiously tailored messaging to promote positive behavioral changes. This exemplifies the potential to expand such initiatives and provide treatment services within this framework.

The discourse presents patterns that are congruent with Islamic principles. These patterns are practicable to implement across a multitude of mental health service and provider relationships to varying degrees. The concept of religious tailoring does not pertain to the imposition of religious beliefs; rather, it focuses on delivering culturally conducive services to Muslim service users, should they find such services beneficial. There is no single religious method for treating mental health, resulting in various approaches (Osman et al. 2021). Some Muslims might have faced past religious or spiritual trauma due to inadequate methods or their previous life circumstances. This entails that service providers and training programs must be prepared to engage the religious and spiritual identities of service users and be aware of any spiritual traumas they may have faced. The degree to which this approach proves advantageous will be contingent upon the individual service user's lived experiences. Likewise, certain Muslims may place greater value on approaches that are more Islamically integrative, while others may prefer therapies that are Western in nature but modified to incorporate Islamic elements. In contrast to Imams, who also occupy a position on this continuum, in that they play a larger role in promoting engagement with Islamic principles to ensure divine contentment. Consequently, our findings shed light on a critical need for differentiation while advocating for the inclusion of diverse approaches. Future research should examine the desirability versus empirical outcomes among Muslim service users in this context.

The limitations of this study include the absence of quantitative analysis. Future research could involve surveys targeting Muslims residing in various provinces and Western regions, using psychometrically validated scales of religiosity. Moreover, while both service users and providers suggested integrating Quran, Hadith, and Prophetic stories, service users provided limited examples of Islamic teachings in practice. Therefore, most Islamic teachings used as examples to illustrate focus group recommendations come from service providers and researchers, including the authors, rather than from service users' views. This gap might have been filled if facilitators engaged further probing during service user focus groups. Still, a deeper exploration of service users' viewpoints on useful Islamic teachings for the recovery process would be very valuable. Future research should look into this by directly asking service users which Islamic teachings they have found helpful for mental health recovery. Lastly, the integration of religious practices within mental health services may encounter resistance from practitioners or users who favor secular methodologies, thereby presenting challenges for effective implementation.

## 6 Conclusion

This study underscores the urgent need for tailored mental health interventions that integrate Islamic values, cultural practices, community support systems, faith‐based counseling, and holistic addiction services to improve service utilization, satisfaction, and treatment outcomes. The study addresses critical deficiencies in Ontario‐based and Muslim community mental health scholarship by incorporating a heterogeneous cohort of participants, integrating perspectives from both service users and providers, collaborating with a community advisory board, and presenting 11 actionable steps for religious tailoring that service providers can implement. Furthermore, this study represents the most extensive qualitative analysis of mental health within the Muslim community to date. This comprehensive methodology aims not only to enhance mental health services but also to foster a deeper understanding of the unique and often invisible challenges faced by Muslim individuals, ultimately promoting greater inclusivity and efficacy in the delivery of mental health services.

## Ethics Statement

The University of Toronto's Research and Ethics Board approved the study, which prioritized participant confidentiality and informed consent. Safeguards included random numerical identifiers, exclusive use of audio recordings, and guidelines to avoid personal identifiers during focus groups. All authors have no conflicts of interest to report.

## Conflicts of Interest

The authors declare no conflicts of interest.

## 1 Peer Review

The peer review history for this article is available at https://www.webofscience.com/api/gateway/wos/peer-review/10.1002/jcop.70036.

## Data Availability

The data that support the findings of this study are available on request from the corresponding author. The data are not publicly available due to privacy or ethical restrictions.
